# Supplementary material for: Programmed magnetic manipulation of vesicles into spatially coded prototissue architectures arrays
Source: Nat Commun. 2020 Jan 13;11:232. doi: 10.1038/s41467-019-14141-x (PMC6957477; doi:10.1038/s41467-019-14141-x)
Supplement: Supplementary file 2 — Supplementary Information [file 41467_2019_14141_MOESM2_ESM.pdf]

Supplementary Information

Programmed magnetic manipulation of vesicles into spatially coded prototissue architectures arrays

Li et al.

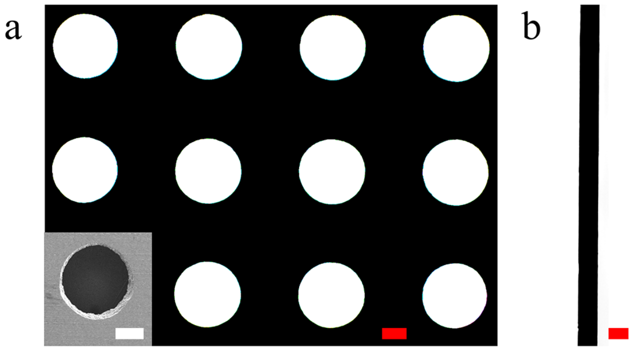

Supplementary Figure 1 Bright field images of the stainless steel mesh with microwell diameter of 250 μm. **a**, Top view. **b**, Side view. The inset is the image of one well taken by scanning electron microscope. The scale bars were 100 μm.

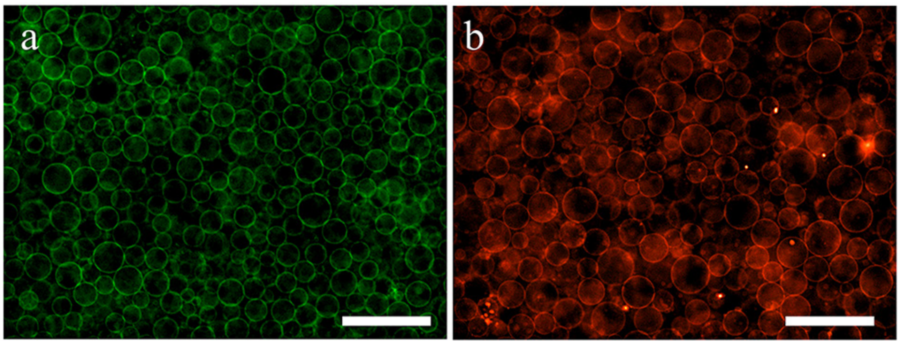

Supplementary Figure 2 Fluorescence images of electroformed GUVs on ITO electrode. **a**, GUVs labelled with NBD PE. **b**, GUVs labelled with TR DHPE. The scale bars were 100 μm.

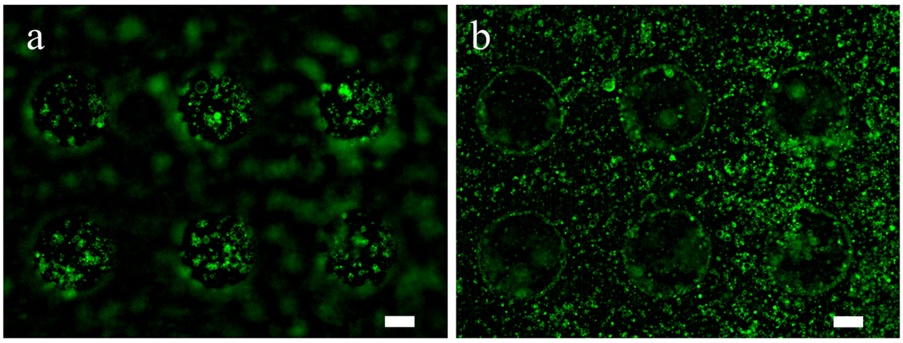

Supplementary Figure 3 Fluorescence images of GUVs around the microwells when no magnetic field was applied. **a**, the image of GUVs in the microwells. **b**, the image of GUVs on the top surface of the SS mesh. No obvious GUVs assembly was observed. The GUVs were homogeneously distributed. The scale bars were 100 μm.

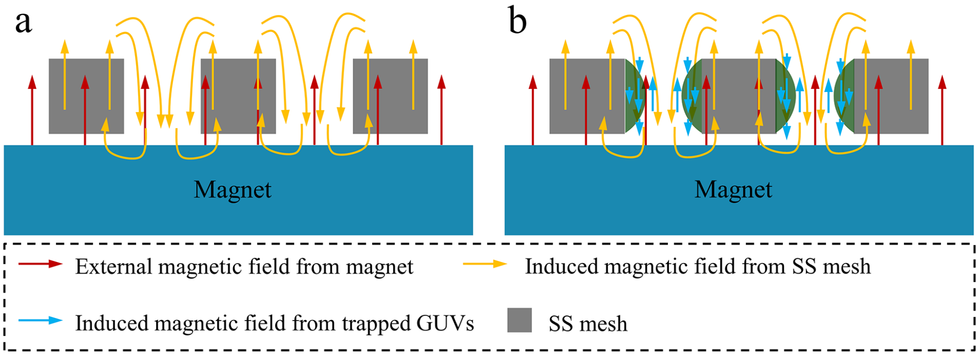

Supplementary Figure 4 Schematic illustration of magnetic field distribution around the SS mesh when no GUVs were trapped (**a**) or the microwells were partially occupied by GUVs colonies (**b**).

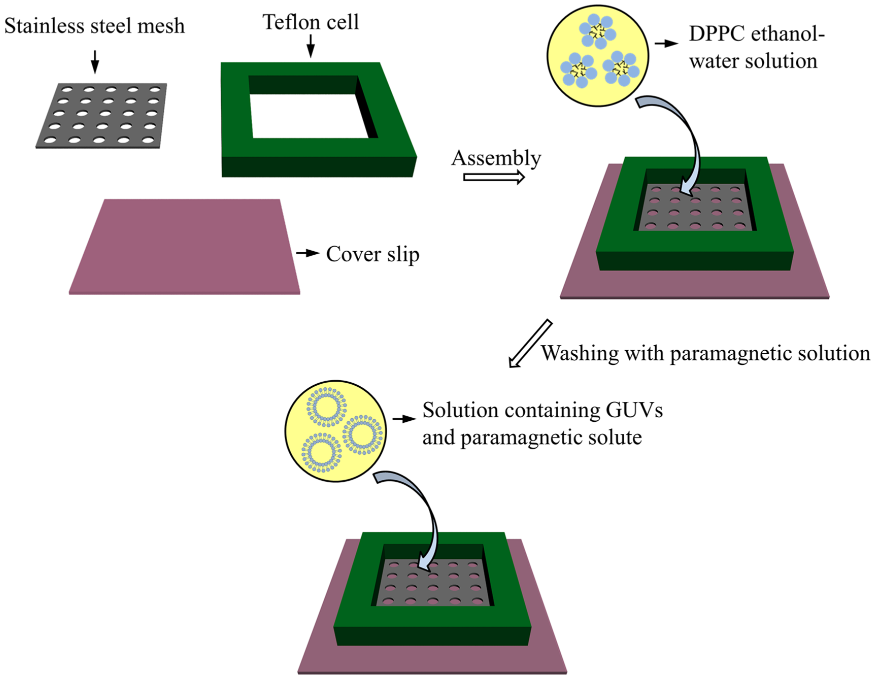

Supplementary Figure 5 Schematic illustration for the assembly of the home-made device for GUVs colonies formation.

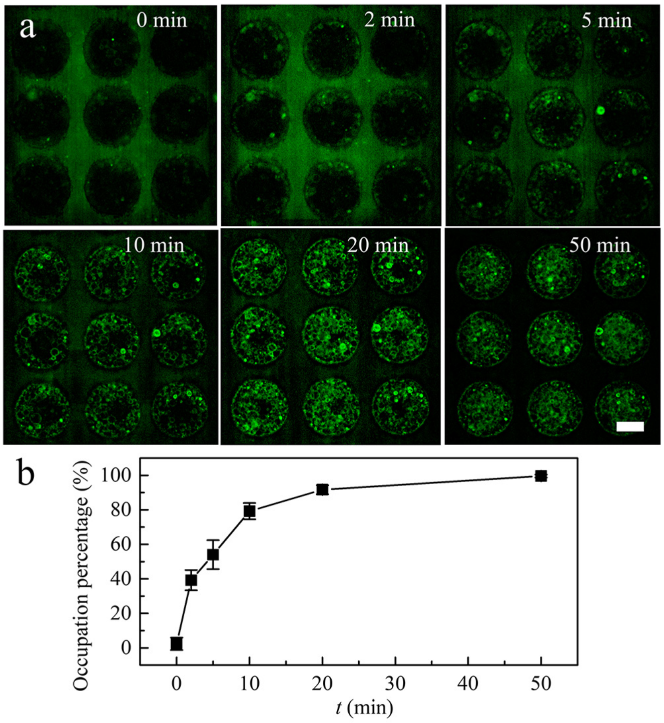

Supplementary Figure 6 Assembly of GUVs in the SS mesh against time under vertical magnetic field. **a**, Fluorescence images against time. **b**, The relationship between occupation percentages against time. Error bars are standard deviations. The scale bar was 100 μm. The error bar represents the standard deviation. Source data are provided as a Source Data file.

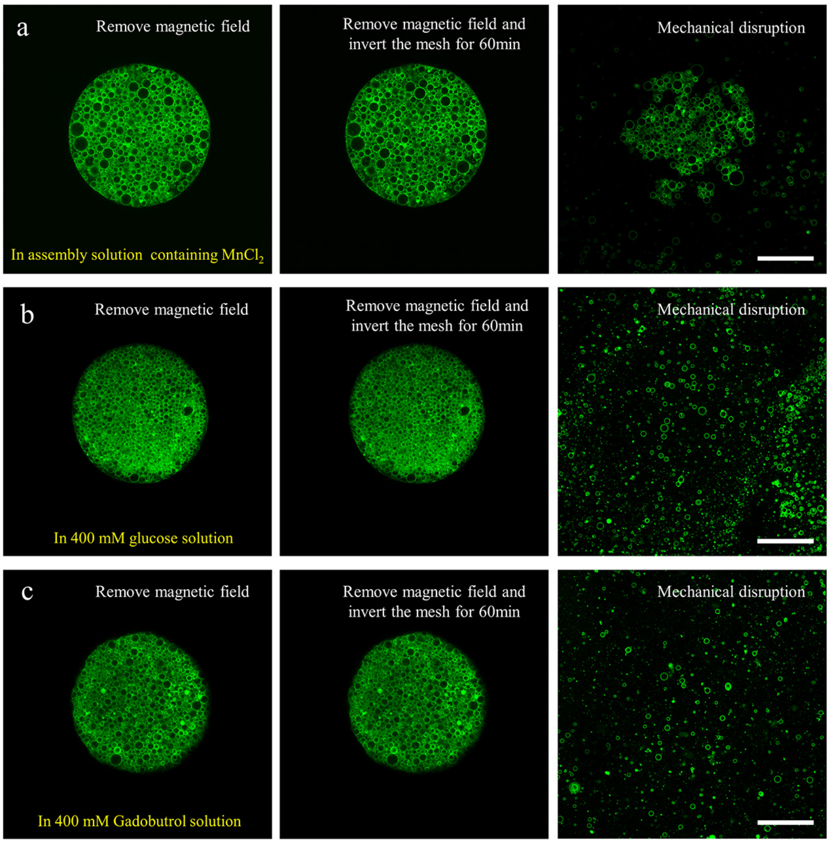

Supplementary Figure 7 Stability of the GUVs assembly. **a**, Fluorescence image of GUVs aggregates in the assembly solution containing $MnCl_2$ after removal of magnetic field (left), being inverted for 60 min with no magnetic field application (middle), and being mechanically disrupted (right). **b**, Fluorescence image of GUVs aggregates in 400 mM glucose solution after removal of magnetic field (left), being inverted for 60 min with no magnetic field application (middle), and being mechanically disrupted (right). **c**, Fluorescence image of GUVs aggregates in 400 mM Gadobutrol solution after removal of magnetic field (left), being inverted for 60 min with no magnetic field application (middle), and being mechanically disrupted (right). The scale bars were 100 μm.

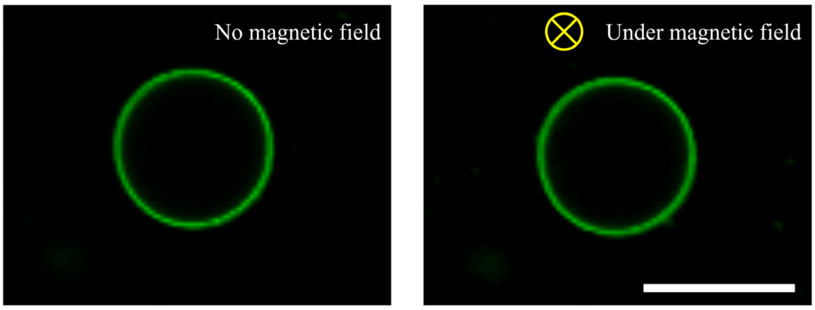

Supplementary Figure 8 Fluorescence images of a single GUV with no application of magnetic field (left) and under ~0.2 T vertical magnetic field (right). The scale bar was 20 μm.

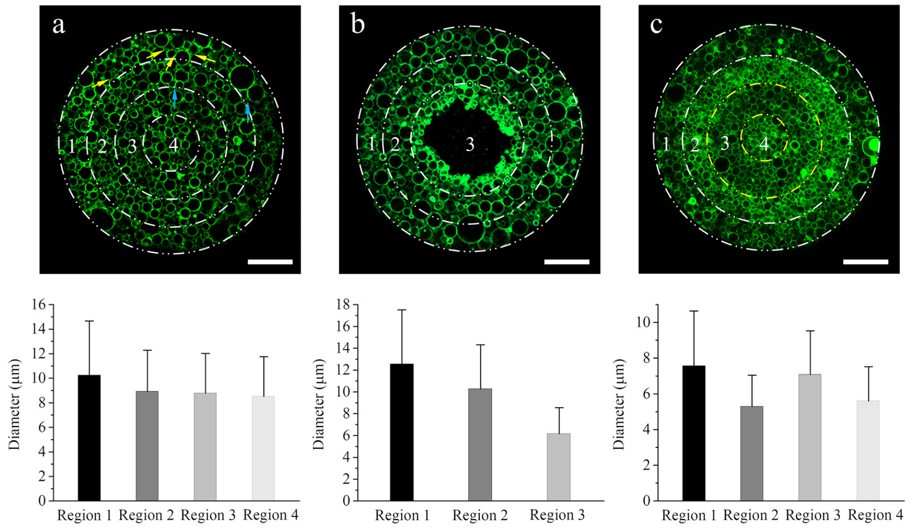

Supplementary Figure 9 Size distribution of GUVs in the microwells. **a**, Fluorescence image of a GUVs colony that fully occupied the microwell (top) and the diagram for the variation of GUVs average size with different regions in the fluorescence image (bottom). The yellow arrows indicated the localization of small GUVs at the voids created by big GUVs. The blue arrows indicated the close packing of big GUVs via deformation and adhesion. **b**, Fluorescence image of a GUVs colony that partially occupied the microwell (top) and the diagram for the variation of GUVs average size with different regions in the fluorescence image (bottom). **c**, Fluorescence image of a GUVs colony via the successive addition of GUVs for two times (top) and the diagram or the variation of GUVs average size with different regions in the fluorescence image (bottom). The scale bars were 50 μm. The error bar represents the standard deviation. Source data are provided as a Source Data file.

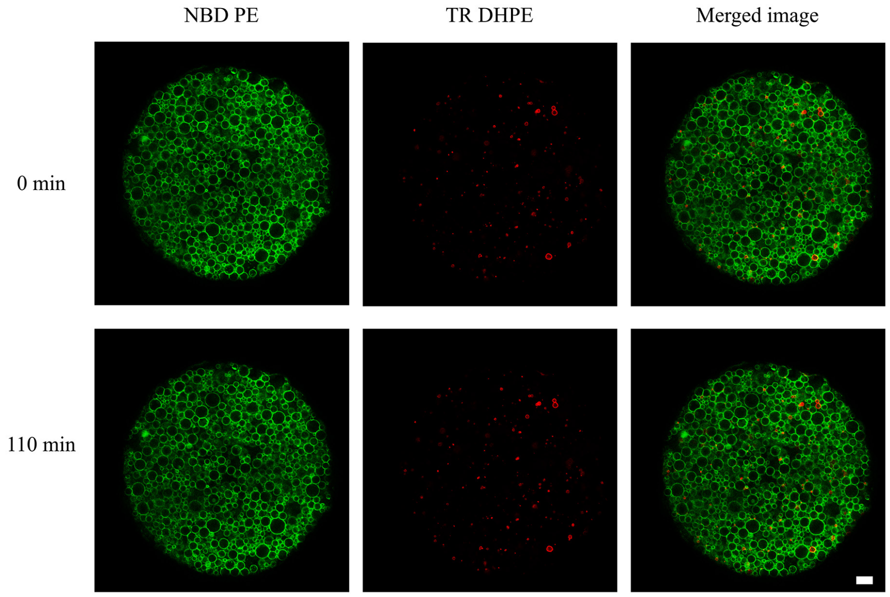

Supplementary Figure 10 Fluorescence images of one GUVs colony composed of GUVs with green fluorescence (labeled with NBD PE) and GUVs with red fluorescence (labeled with TR DHPE) at 0 min and 110 min. The scale bar was 20 μm.

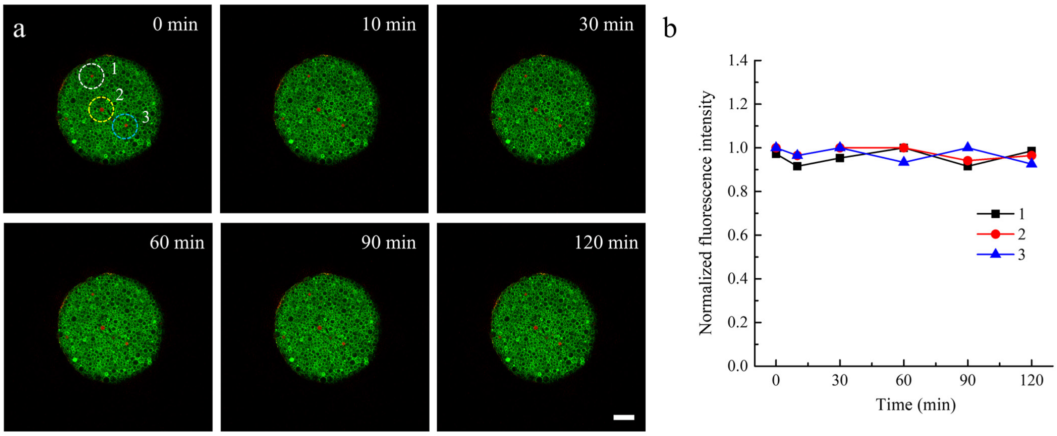

Supplementary Figure 11 Integrity study of the compartmentalized interior of GUVs in the colony. **a**, Merged fluorescence image of a GUVs (labeled with green NBD PE) colony containing some GUVs encapsulated with Rhodamine B isothiocyanate-Dextran (molecular weight, 70 kDa, red fluorescence). Three GUVs encapsulated with Rhodamine B isothiocyanate-Dextran were presented in the dash circles. **b**, Normalized fluorescence intensity against time of the three GUVs in **a**. The scale bar was 50 μm. Source data are provided as a Source Data file.

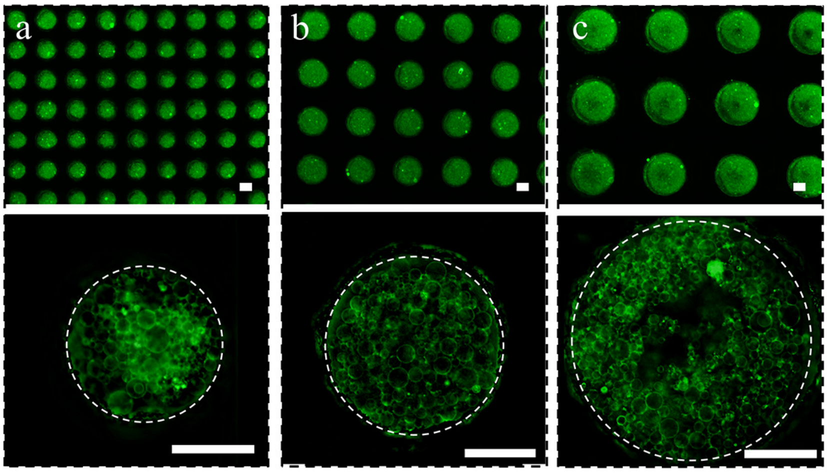

Supplementary Figure 12 Modulation of the size of the GUVs colonies via the trapping experiments on the SS mesh with different microwell diameters: 150 μm (**a**), 250 μm (**b**), and 350 μm (**c**). The dash circles indicated the microwell walls. The scale bars were 100 μm.

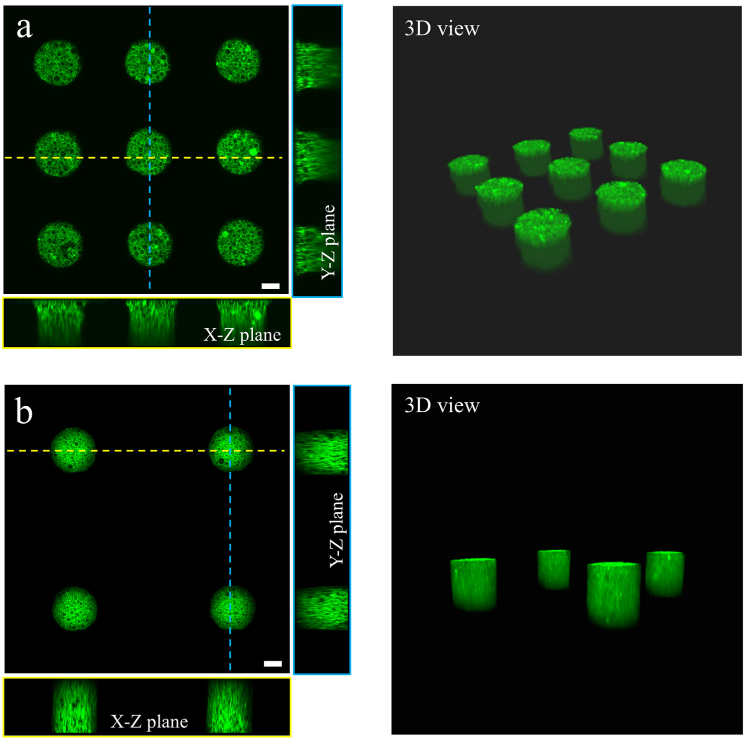

Supplementary Figure 13 Fluorescence images of GUVs colonies with 3D aspect ratio of ~1 (**a**) and ~1.5 (**b**). The yellow dash line indicated the section line for X-Z profile. The blue dash line indicated the section line for Y-Z profile. The scale bars were 100 μm.

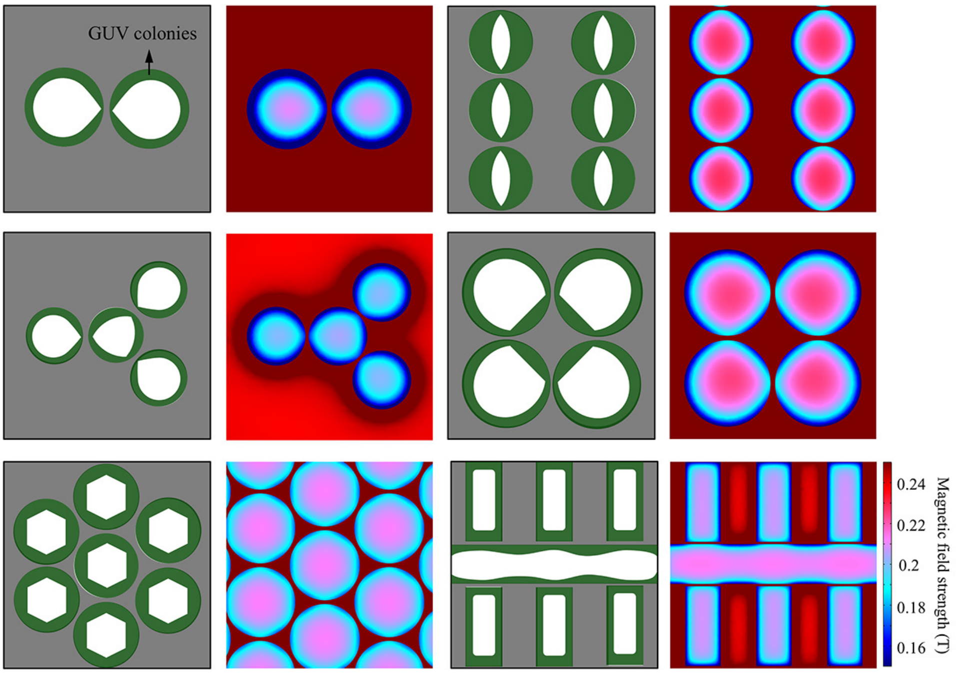

Supplementary Figure 14 Predicted morphologies of GUVs colonies under vertical magnetic field through simulation: round colonies with oval, elliptical, heart-shaped, half-round, or hexagonal holes, and striped colonies with waved edges.

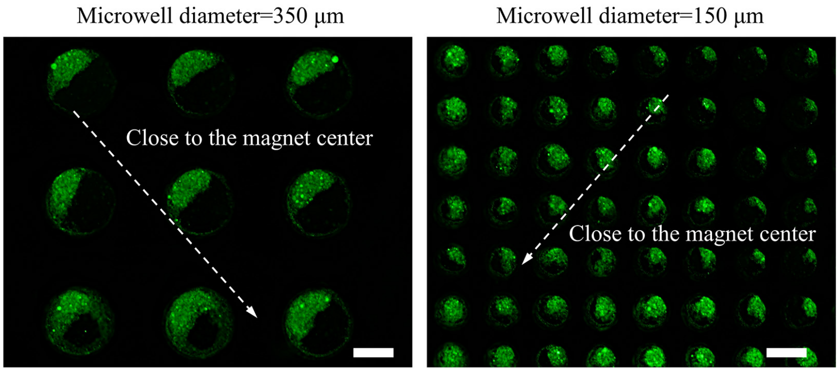

Supplementary Figure 15 Fluorescence images of the graded asymmetric colonies formed in microwells with diameter of 350 μm and 150 μm under inclined magnetic field. The scale bars were 200 μm.

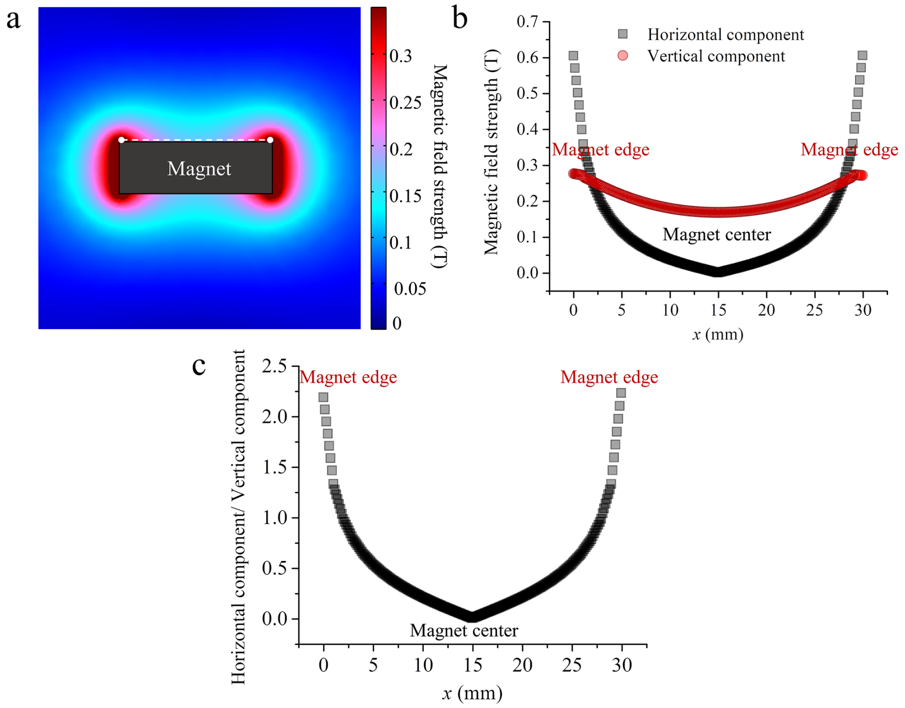

Supplementary Figure 16 Simulated magnetic field distribution around a magnet. **a**, magnetic field distribution along the longitudinal section of the magnet across the magnet center. **b**, Simulated horizontal component and vertical component of the magnetic field along the white dash line in (**a**). **c**, Simulated ratio of horizontal component to vertical component of the magnetic field along the white dash line in (**a**).

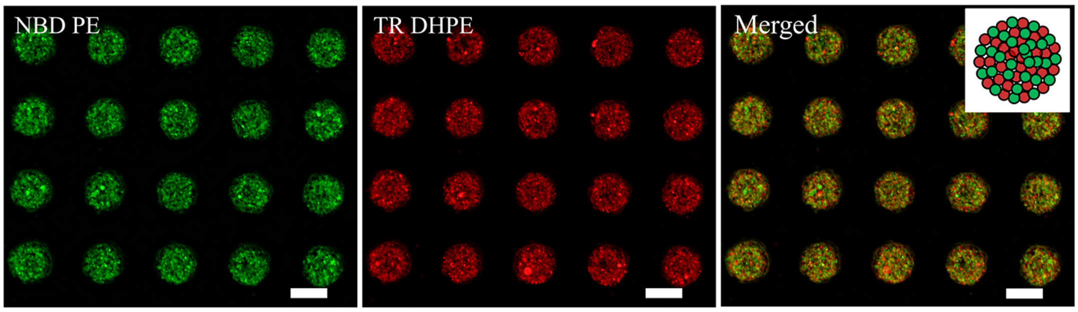

Supplementary Figure 17 Fluorescence images of parallelly coded GUVs colony arrays formed under vertical magnetic field. The scale bars were 200 μm.

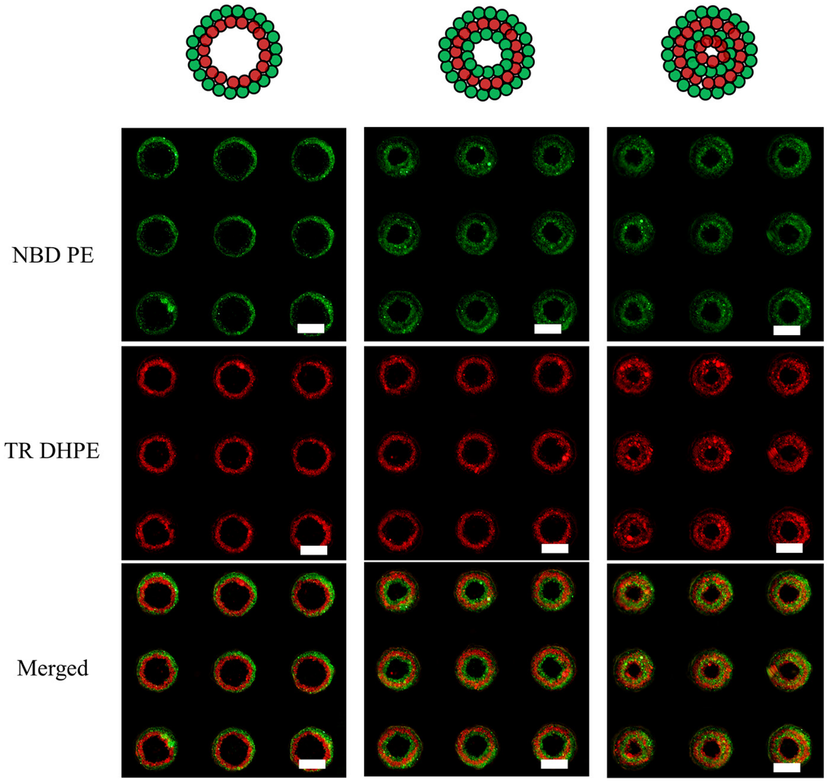

Supplementary Figure 18 Schematics and fluorescence images coaxially serial coded GUVs colony arrays. The scale bars were 200 μm.

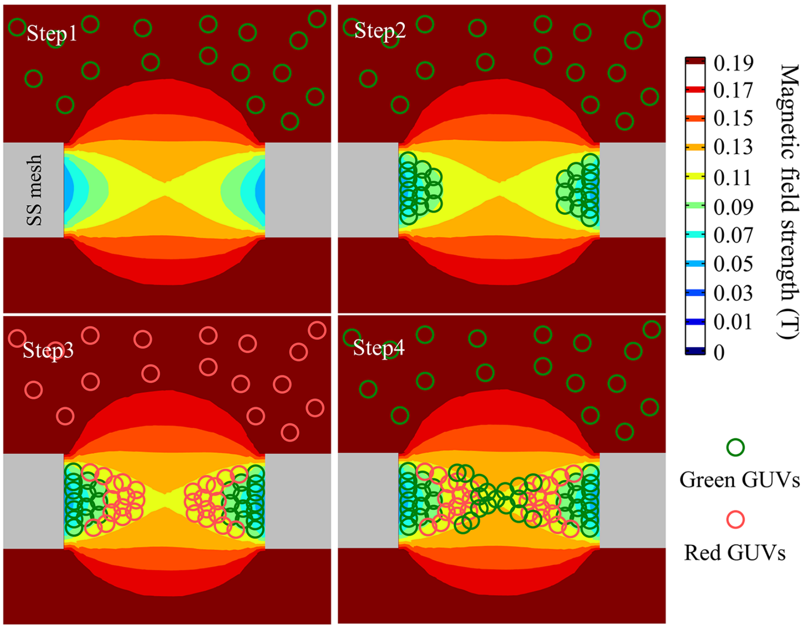

Supplementary Figure 19 Schematic illustration of the assembly of coded GUVs in the microwells guided by magnetic field.

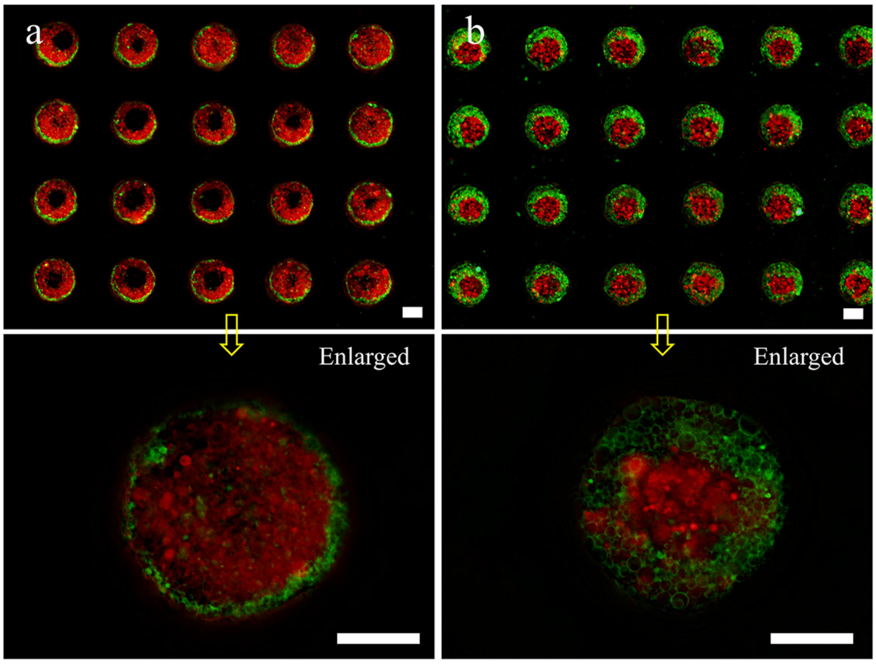

Supplementary Figure 20 Fluorescence images of coaxial hybrid "colonies" containing two layers of GUVs under vertical magnetic field. **a**, Less green GUVs. **b**, More green GUVs. The scale bars were 100 µm.

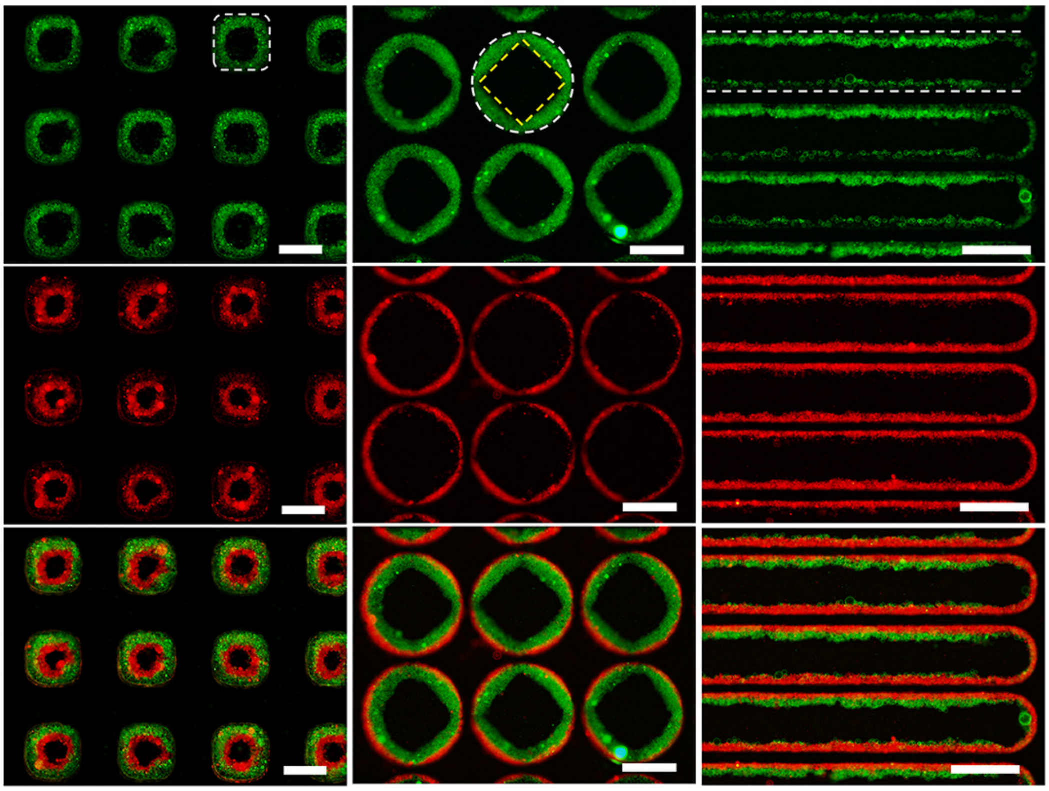

Supplementary Figure 21 Formation of serially coded GUVs colony arrays with other morphologies under vertical magnetic field. The scale bars were 200 μm. The white dash rectangle, circle and line illustrated rough outline of the GUVs colonies. The yellow dash box in the bottom image indicated square hole of the GUVs colony.

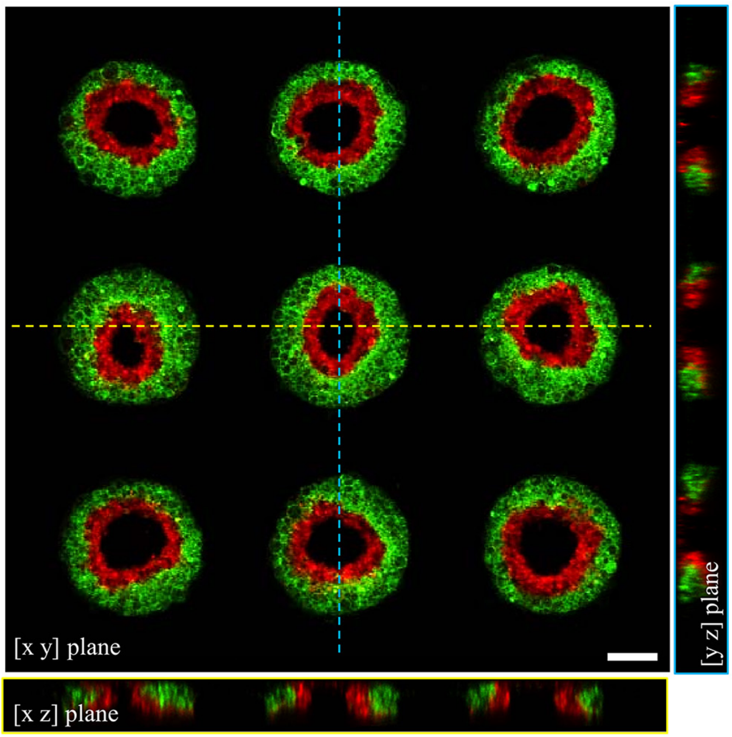

Supplementary Figure 22 Fluorescence images of the microstructures composed of two coaxial GUVs colonies at different planes taken by laser confocal microscope. The yellow dash line indicated the section line for X-Z profile. The blue dash line indicated the section line for Y-Z profile. The scale bar was 100 μm.

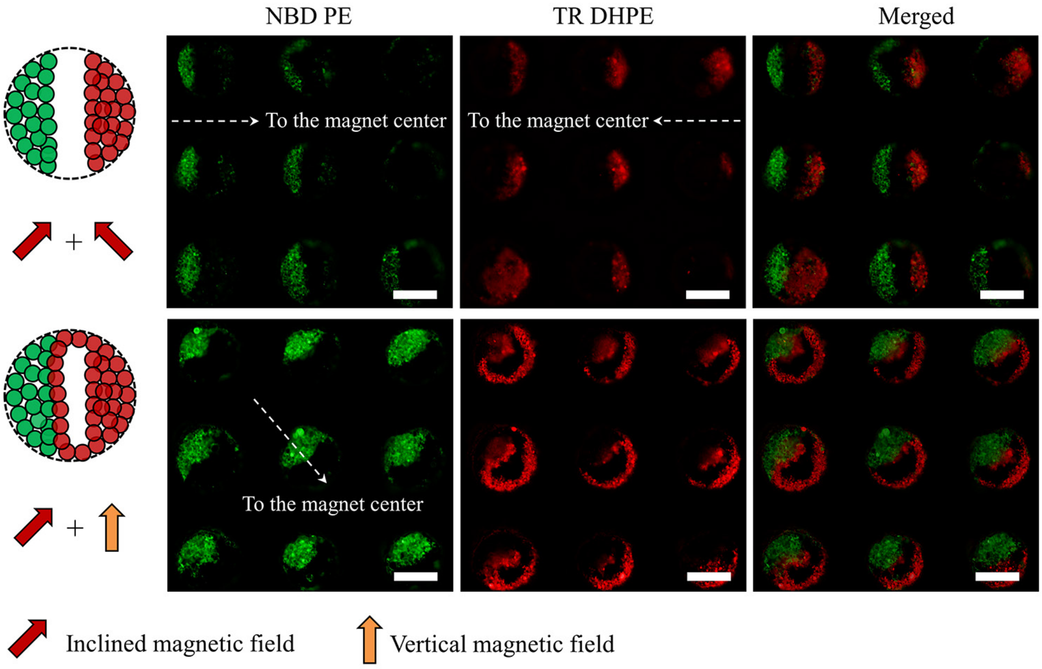

Supplementary Figure 23 Schematics and fluorescence images of asymmetric GUVs colonies formed via the successive application of two intermediate magnetic fields with different directions (top) and one intermediate magnetic field and another vertical magnetic field (bottom). The scale bars were 200 μm.

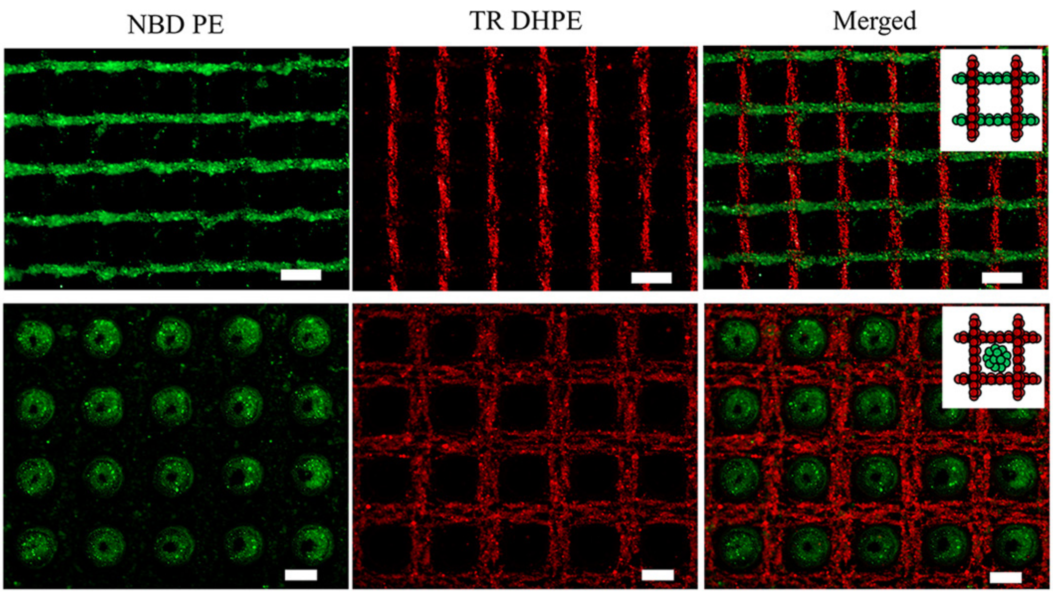

Supplementary Figure 24 Fluorescence images of mesh-like GUVs colonies under horizontal + horizontal magnetic field (top) and vertical + horizontal + horizontal magnetic field (bottom). The scale bars were 200 µm.

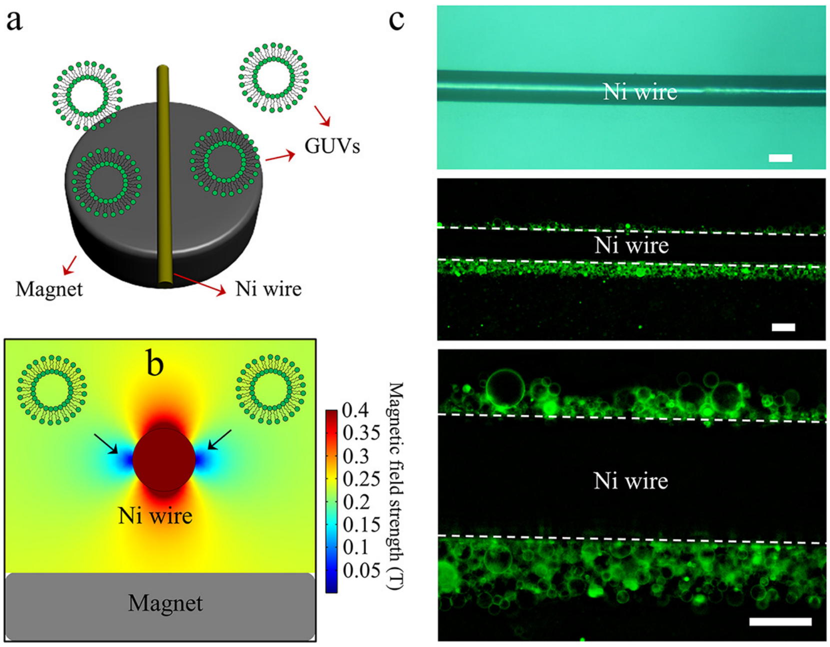

Supplementary Figure 25 Assembly of GUVs colonies around nickel wire under vertical magnetic field. **a**, The schematic for the device. **b**, Simulated magnetic field distribution around a nickel wire on the magnet. **c**, Bright field image of a nickel wire (top) and the fluorescence images of GUVs colonies around the nickel wire (bottom). The scale bars were 100 µm.

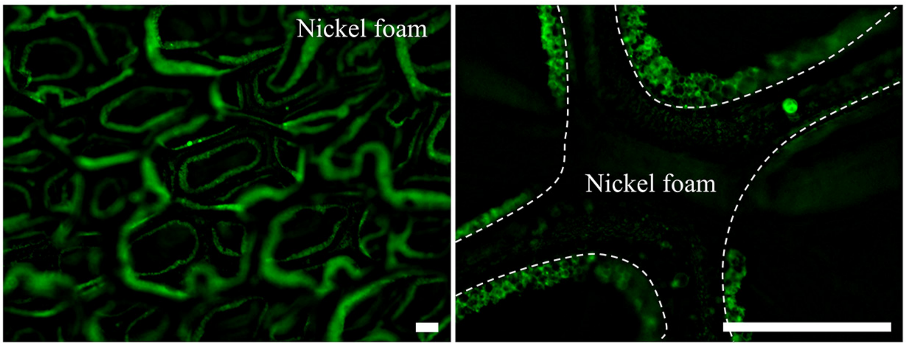

Supplementary Figure 26 Fluorescence images of GUVs colonies formed around nickel form under vertical magnetic field. The scale bars were 500 μm.

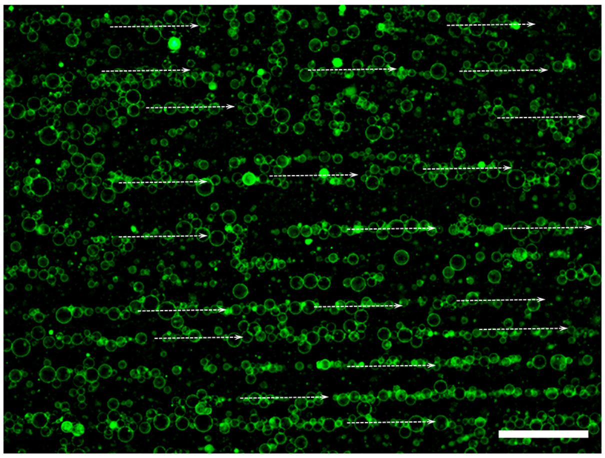

Supplementary Figure 27 Fluorescence image of the GUVs assemblies formed on stainless sheet with scratches. The scale bars were 200 μm.

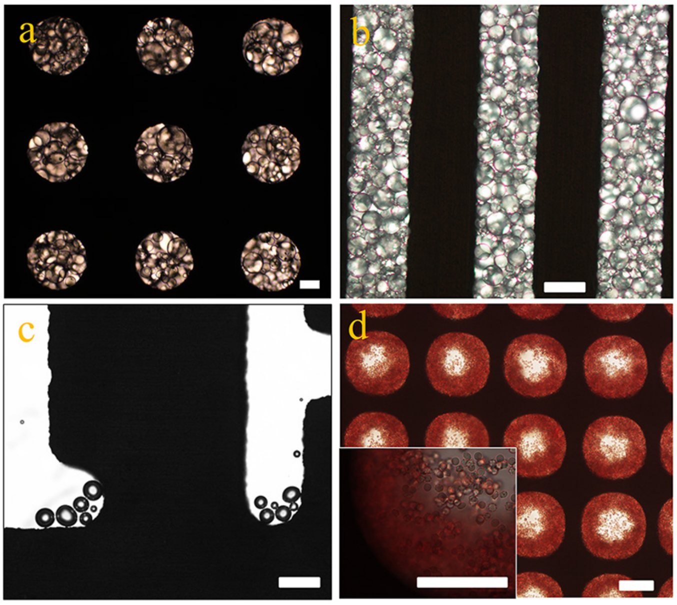

Supplementary Figure 28 Assembly of other materials in the microwells. **a**, Sephadex. **b**, Oil droplet. **c**, Bubbles. **d**, Erythrocytes. The scale bars were 100 μm.

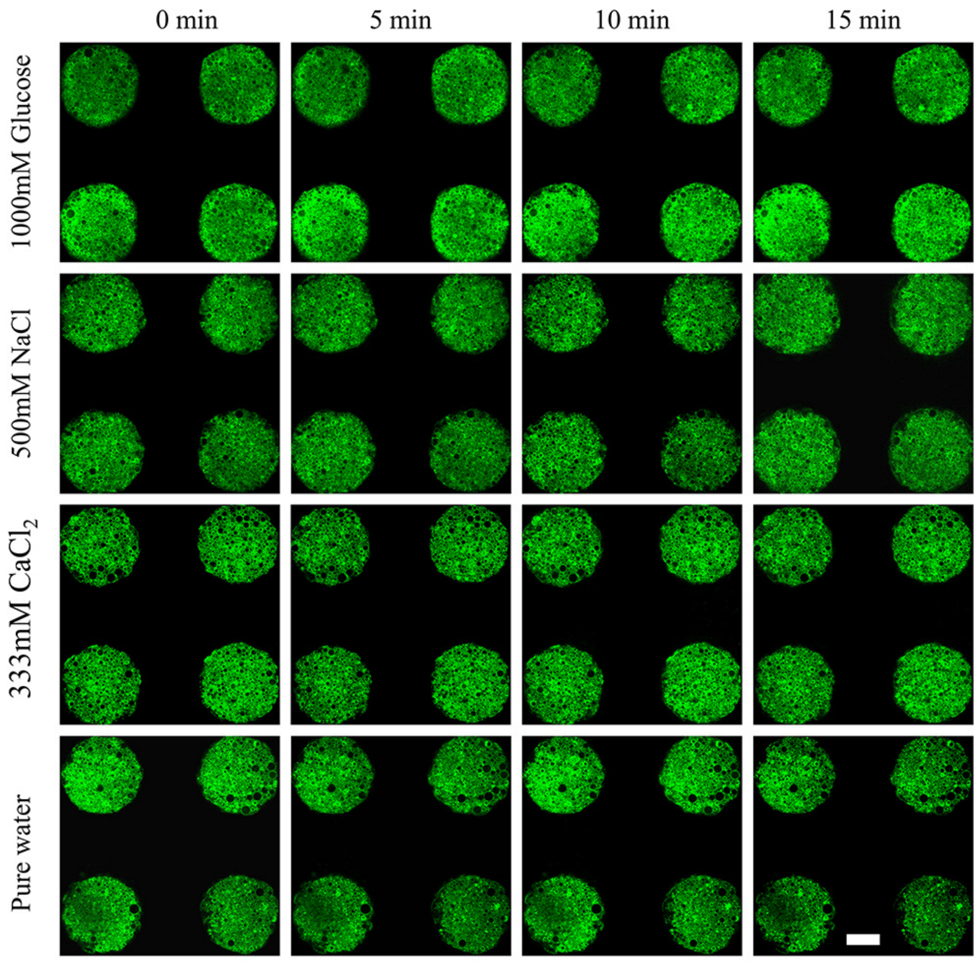

Supplementary Figure 29 Fluorescence images of the GUVs colonies with time in different unbalanced osmotic conditions. The scale bar was 100 μm.

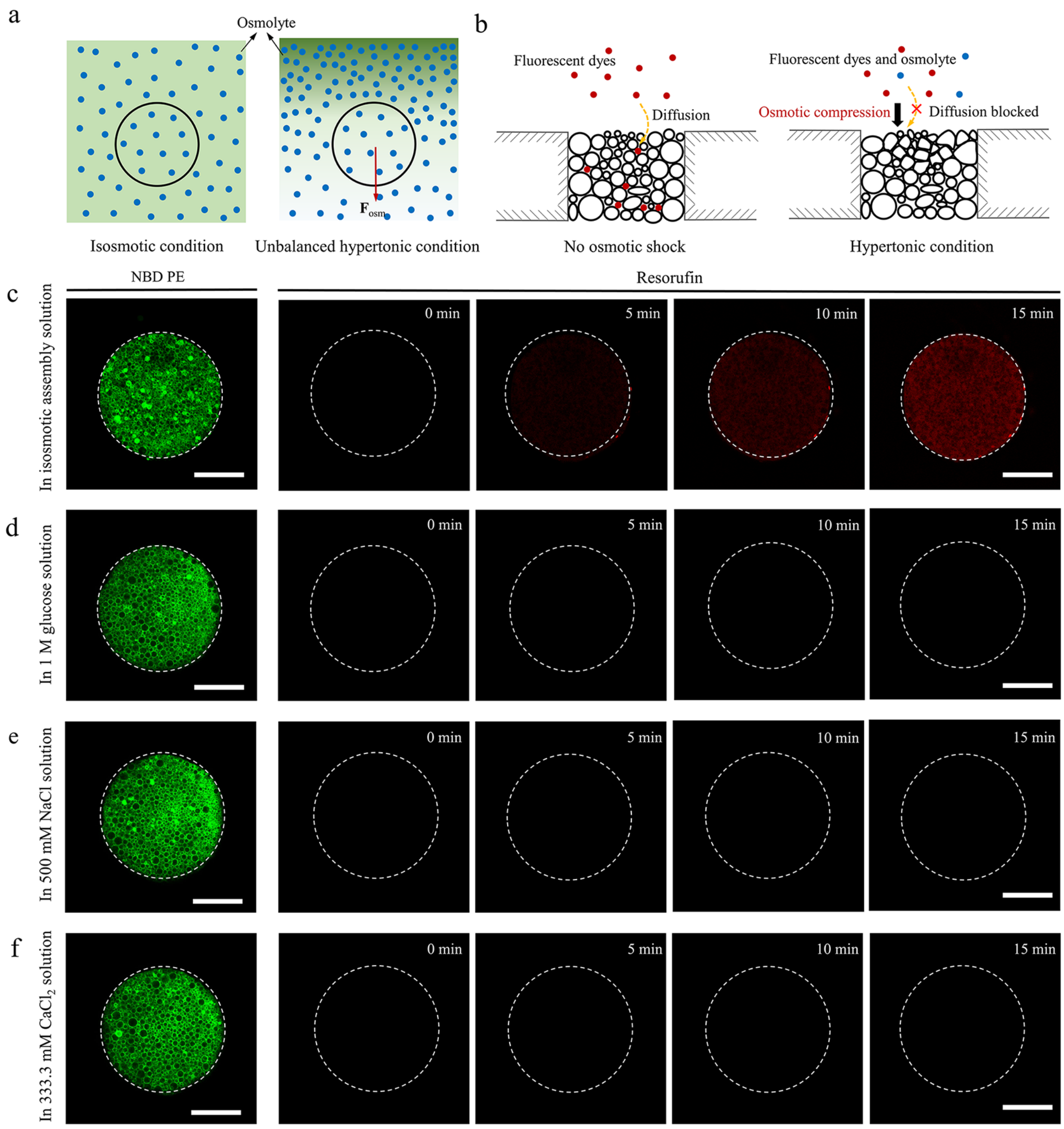

Supplementary Figure 30 Mechanism of the GUVs colony's osmotic stability. **a**, Schematic of GUV in isosmotic condition and unbalanced hypertonic condition. **b**, Schematic of GUVs colony in isosmotic condition and hypertonic condition. **c**, Fluorescence images illustrating the diffusion of resorufin (red fluorescence) into the voids of GUVs colony (green fluorescence) in isosmotic assembly solution. **d-f**, Fluorescence images illustrating the failure of resorufin to enter in the GUVs colony voids in hypertonic 1 M glucose solution (**d**), 500 mM NaCl solution (**e**), and 333.3 mM $CaCl_2$ solution (**f**). The scale bars were 100 μm.

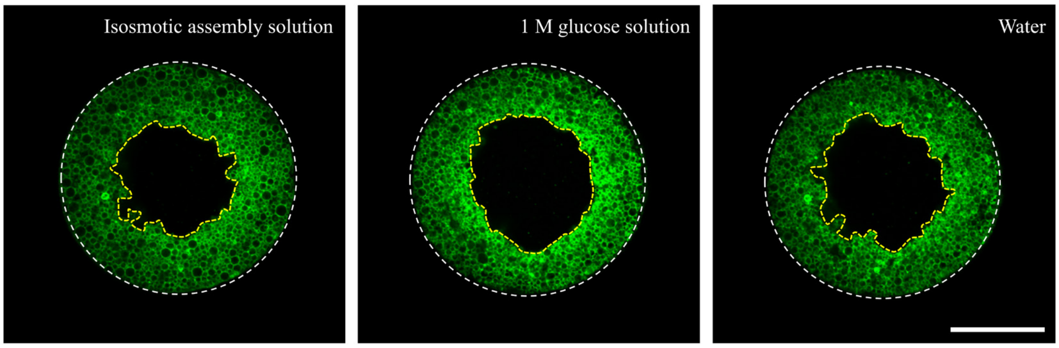

Supplementary Figure 31 Fluorescence images of GUVs colony that partially occupied the microwell successively in isosmotic assembly solution containing $MnCl_2$ (left), 1 M glucose solution (middle), and pure water (right). The white dash circle indicated the microwell wall. The yellow dash line indicated the boundary between GUVs colony and surrounding solution. The scale bars were 100 µm.

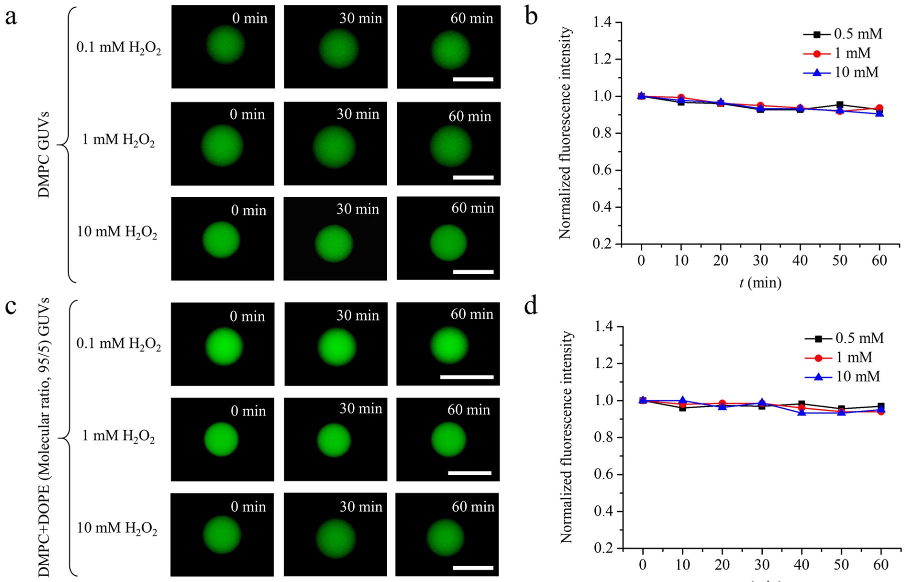

Supplementary Figure 32 Influence of hydrogen peroxide on GUVs membrane permeability. **a**, Fluorescence images of DMPC GUVs containing calcein against incubation time in hydrogen peroxide solution with different concentrations. **b**, Variation of the normalized fluorescence intensity of calcein in DMPC GUVs against incubation time in hydrogen peroxide solution with different concentrations. **c**, Fluorescence images of DMPC/DOPE (Molecular ratio, 95/5) GUVs containing calcein against incubation time in hydrogen peroxide solution with different concentrations. **d**, Variation of the normalized fluorescence intensity of calcein in DMPC/DOPE (Molecular ratio, 95/5) GUVs against incubation time in hydrogen peroxide solution with different concentrations. The scale bars were 20 µm. Source data are provided as a Source Data file.

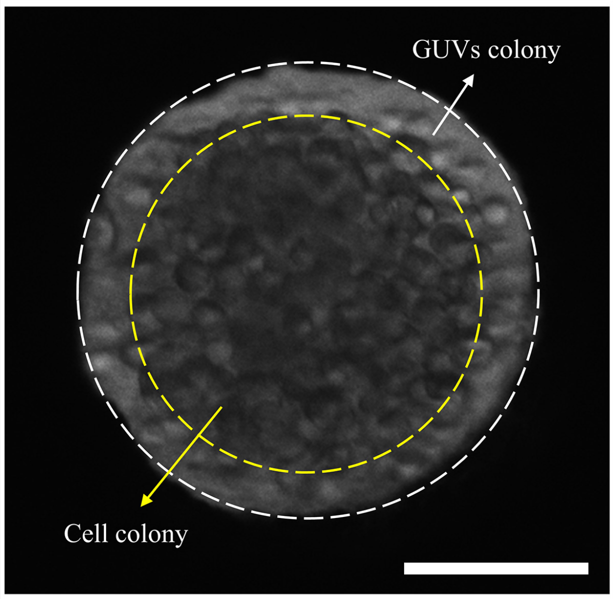

Supplementary Figure 33 Bright field image of one unit of the array with coaxial GUVs colony and cell colony. The scale bar was 100 μm.

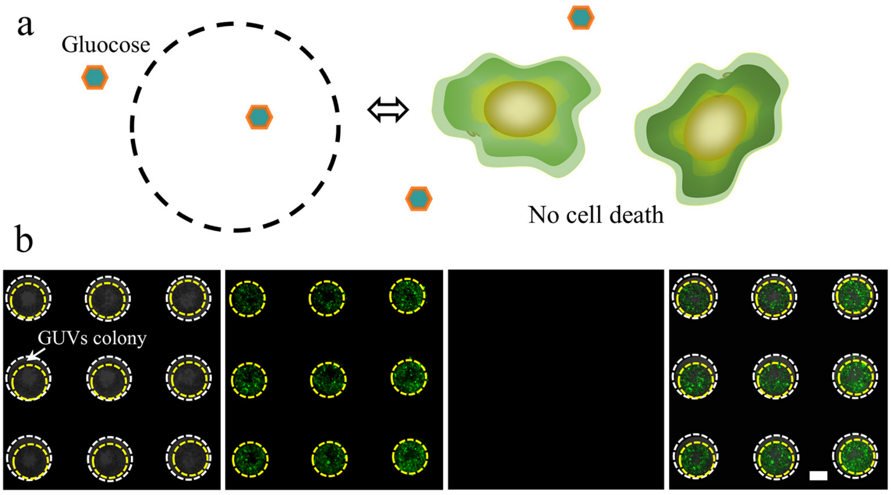

Supplementary Figure 34 Communication between GUVs colonies with no GOD and cell colonies. **a**, The schematic. **b**, Images of the colonies array after the incubation of cells with GUVs for 6h: bright field image (the first one), fluorescence image of live cells stained by fluorescein diacetate (the second one), fluorescence image of dead cells stained by propidium iodide (the third one), and merged image (the last one). No cell death was observed in the sample.
