# Supplementary material for: Programmed magnetic manipulation of vesicles into spatially coded prototissue architectures arrays
Source: Nat Commun. 2020 Jan 13;11:232. doi: 10.1038/s41467-019-14141-x (PMC6957477; doi:10.1038/s41467-019-14141-x)
Supplement: Supplementary file 4 — Description of Additional Supplementary Files [file 41467_2019_14141_MOESM4_ESM.pdf]

Title: Supplementary Movie 1.

Description: Monitoring of one GUVs colony composed of gGUVs and rGUVs with time.
